# Cardiac Axis in Early Gestation and Congenital Heart Disease

**DOI:** 10.2174/011573403X264660231210162041

**Published:** 2024-01-24

**Authors:** D. Carrasco, L. Guedes-Martins

**Affiliations:** 1 Instituto de Ciências Biomédicas Abel Salazar, University of Porto, 4050-313 Porto, Portugal;; 2 Centro de Medicina Fetal, Medicina Fetal Porto, Serviço de Obstetrícia - Centro Materno Infantil do Norte, Porto 4099-001, Portugal;; 3 Centro Hospitalar Universitário do Porto EPE, Centro Materno Infantil do Norte, Departamento da Mulher e da Medicina Reprodutiva, Largo Prof. Abel Salazar, 4099-001 Porto, Portugal;; 4 Unidade de Investigação e Formação-Centro Materno Infantil do Norte, 4099-001 Porto, Portugal;; 5 Instituto de Investigação e Inovação em Saúde, Universidade do Porto, Porto, 4200-319, Portugal

**Keywords:** Doppler, fetal echocardiography, four-chamber, congenital heart defect, fetal cardiac axis, abnormal fetal cardiac axis

## Abstract

Congenital heart defects represent the most common structural anomalies observed in the fetal population, and they are often associated with significant morbidity and mortality.

The fetal cardiac axis, which indicates the orientation of the heart in relation to the chest wall, is formed by the angle between the anteroposterior axis of the chest and the interventricular septum of the heart. Studies conducted during the first trimester have demonstrated promising outcomes with respect to the applicability of cardiac axis measurement in fetuses with congenital heart defects as well as fetuses with extracardiac and chromosomal anomalies, which may result in improved health outcomes and reduced healthcare costs.

The main aim of this review article was to highlight the cardiac axis as a reliable and powerful marker for the detection of congenital heart defects during early gestation, including defects that would otherwise remain undetectable through the conventional four-chamber view.

## 1 INTRODUCTION

Congenital heart defects (CHD), which are the most common birth defects, occur when the fetal heart does not develop normally during pregnancy [1]. CHD can be categorized as either structural or functional anomalies of the heart and/or major blood vessels that are present at birth [2]. The worldwide incidence of CHD has been progressively increasing, and congenital ventricular and atrial septal defects represent more than two-thirds of all types of CHD [3-5]. Frequently, the symptoms of CHD become apparent only after birth, with the closure of the fetal shunts [6].

Most cases of CHD are multifactorial and related to familial, maternal, or even fetal conditions [7, 8]. Nevertheless, the exact underlying causes of CHD are still uncertain, and a considerable number of affected children are born without any identifiable risk factors for CHD [7, 8].

CHD are a significant public health concern, and in neonates, severe forms of CHD can severely impact the quality of life and even be life-threatening [2].

CHD are the leading cause of morbidity, medical expenses, and death due to congenital malformations during the first year of life [1, 9]. The incidence of CHD varies widely across the world due to disparities in economic factors and health facilities [10]. While most CHD are minor, 25% of newborns suffer from severe forms of cardiac abnormalities and have a poor prognosis [11]. Depending on the severity, CHD may require early interventions, multiple hospitalizations, and often lifelong care, which contribute to high costs [12].

Remarkable achievements in cardiovascular medicine, anesthesia, pediatric care, and surgery have drastically decreased the mortality and morbidity rates of CHD, making the global crude mortality rate decline to 2.8 per 100,000 in 2019. This has enabled most CHD patients to reach adulthood [13, 14].

A wide range of studies have shown that increased investment in detecting CHD during early life stages can improve life outcomes and lower health care costs [15]. It can also allow parents to make informed decisions regarding the pregnancy; should they wish to terminate the pregnancy, it can be done at an earlier time and in a safer way. If the pregnancy is continued, the timing, location, mode of delivery, and direct postnatal care can be thoroughly arranged [16].

The ideal gestational age for screening for CHD is between 18 and 22 weeks [17]. Image resolution at an early stage in gestation can be technically challenging due to multiple fetal movements and reduced image resolution, as well as the small size of the cardiac structures [6, 17]. However, detailed segmental evaluations are still achievable in most fetuses. In fact, reports of successful fetal echocardiography have been described for over 30 years in the first trimester [18-21]. Since then, multiple advances in imaging technology, knowledge of fetal embryology, and the application of new ultrasound markers have expanded the capacity of fetal echocardiograms and fueled increasing interest in early anomaly detection [22-24]. The use of high-resolution ultrasonography has recently enabled a more comprehensive examination of fetal anatomy, resulting in the ability to detect a greater variety of abnormalities during the first trimester than was previously possible [25].

Several indirect markers, including increased nuchal translucency (NT), tricuspid regurgitation, and reverse flow in the ductus venosus, have been shown to enhance the detection rate of hidden cardiopathy [26-30]. Additionally, the fetal cardiac axis (CAx) has been recognized as a potentially valuable tool for detecting CHD during early gestation [26]. In fact, previous studies have shown that an abnormal CAx is present in a significant percentage of fetuses with CHD in the first trimester [31].

This review aimed to offer a comprehensive understanding of the potential advantages of incorporating fetal CAx measurements in identifying CHD during early gestation, which may result in improved health outcomes and reduced healthcare costs.

## 2 METHODS

This review was conducted by searching the PubMed database using relevant keywords, such as “Doppler,” “fetal echocardiography,” “congenital heart defect,” “fetal cardiac axis”, “four-chamber view”, and “abnormal fetal cardiac axis”.

The search terms were used alone and in combination with each other.

In the preselection process, only scientific publications that were related to the topic (based on the title), published within the last 38 years and written in English, were included. The complete texts of the selected articles were retrieved for thorough analysis.

**Fig. (1):**
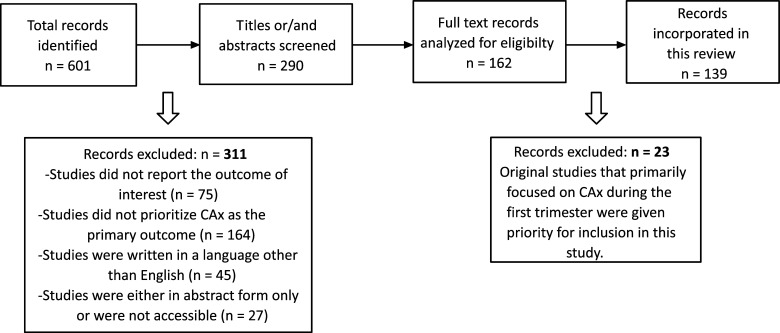
Flowchart of the search results.

The final selection of articles included in this review was based on the overall quality and rigor of each article with respect to the research question (Fig. **1**).

## 3 CHANGES IN THE FETAL CARDIAC AXIS DURING THE FIRST TRIMESTER OF PREGNANCY

The heart is the embryo's first functional organ to develop, and throughout evolution, the general sequence of events that determine how the heart develops has been substantially preserved in all vertebrates, involving complex biological signals, specification of myocardial progenitor cells, and heart tube looping [32, 33]. Understanding the various stages of early cardiogenesis and the process of fetal CAx rotation may lead to a better comprehension of the CAx and a more appropriate classification and prognosis of CHD [34].

Cardiac development is initiated at gastrulation, at the end of the second week of human development, emerging from the mesodermal embryonic germ layer [35]. Early in embryogenesis, a single heart tube is created when the left and right wings of the heart primordium unite across the midline [36]. Subsequently, at approximately day 23 of development, the heart begins to contract rhythmically while it is still a singular linear tube, and active fetal circulation is established by the end of the fourth week [33, 35, 37]. Through complex cellular interactions and heart looping in utero, the 4-chambered heart is formed [32, 33, 35, 38]. By the eighth week of gestation, the atria, ventricles, venous connections, arterial roots, and intrapericardial arterial trunks are often fully formed [35, 39, 40]. Furthermore, fetal CAx rotation, which is detectable in the late first trimester, is believed to occur during the heart's ultimate phase of embryogenesis; this phase involves heart tube looping, which is characterized by a manifold of positional changes [36, 41, 42]. In fact, a successful looping stage, *i.e*., where the developing heart initially forms a straight tube and then undergoes rightward looping, may be crucial for the proper alignment of the future heart chambers and tracts [43, 44]. Any perturbation in the correct looping of the cardiac tube can lead to an abnormal axis, which may increase the likelihood of CHD, particularly those involving the outflow tracts [43, 44]. Thus, over the past few decades, to gain a more comprehensive understanding of cardiac axis formation and dynamic morphogenesis, a considerable amount of research has been conducted to understand the patterns of heart tube formation and looping [32, 33].

At 4 weeks of gestation, cardiac looping begins; this very complex process includes geometrical changes with bending and torsional configuration, and it is regulated by both mechanical forces and genetic factors [42]. For example, paired-like homeodomain transcription factor 2 (PITX2) and basic helix-loop-helix (bHLH) transcription factor (eHAND) are expressed during cardiac looping [45]. Additional transcription factors, including GATA binding protein 5 (GATA5) and GATA binding protein 6 (GATA6), are predominantly located at the ends of the cardiac tube [45]. However, the roles of mechanical and genetic factors in regulating cardiac looping have yet to be fully elucidated, and further studies are required to gain a deeper understanding of the underlying mechanisms.

During the initial stages of looping, the ventricular relationship is craniocaudal [41]. Later, at week 4, the heart tube undergoes rightward looping, where the ventricles bend and twist, and the right ventricle assumes a rightward, dorsal posture relative to the left ventricle [41, 42]. During the final stage of ventricular looping, the right ventricle moves anteriorly as the heart “untwists” anticlockwise around the basoapical axis [46, 47]. Throughout this process, the heart tube increases fivefold in length as a result of the constant inclusion of newly differentiated cardiomyocytes [35]. Evidence suggests that this 'untwisting' phase of looping and the late developmental modifications in right ventricular morphology may be accountable for the modification of the cardiac axis observed during early fetal echocardiography. As a result, midline hearts that do not complete this final embryological stage exhibit both a shift in the CAx and an aberrant apical shape [41].

Currently, it is unknown which specific embryologic event causes some fetuses to have an abnormal CAx. However, studies have suggested that excessive rotation of the bulboventricular loop during early development may result in left-axis deviation (large axis), whereas incomplete rotation could lead to a right-axis deviation (small axis), both of which may underlie an abnormal CAx [48]. As an example, conotruncal anomalies (such as tetralogy of Fallot) have been reported to contribute to a left-axis deviation [41]. Additionally, certain conditions may cause a size disproportion of the heart chambers (dilation/hypoplasia), further contributing to this anomaly [48, 49]. As an example, hypoplastic left heart syndrome has been associated with significant leftward deviation of the CAx [50]. Another factor to consider is that CAx deviation may also arise from abnormal pressure resulting from an underdeveloped abdominal wall or diaphragm, such as a diaphragmatic hernia [51-54].

The fetal CAx, which indicates the orientation of the heart in relation to the chest wall, is formed by the angle between the anteroposterior axis of the chest (defined as a line passing across the sternum and anterior spinous process) and the interventricular septum of the heart [55].

Accurate assessment and evaluation of the CAx may be crucial during a cardiac examination, as irregular deviations of the CAx have been reported in fetuses with long-term cardiovascular health complications, including CHD [48, 56, 57]. To ensure accurate measurement of the CAx, reliable methods have been well-defined for use during the mid and third trimesters [58].

There is some inconsistency in the provided data regarding the normal values of the CAx during early gestation. This discrepancy could be attributed to several factors, including the small number of patients in earlier studies, technological difficulties, and the poor quality of cardiac imaging before 12 weeks of pregnancy [41]. Therefore, with the development of high-resolution ultrasonography, some research has been done to establish reference values for CAx measurement in early gestation [26, 41, 50, 57].

Earlier in pregnancy, at 8 weeks, the embryonic CAx has a more midline orientation [41]. Then, toward the end of the first trimester, the heart's position in the chest rotates gradually to the left, with a slight rotation of the apex, changing from 39° at 11 weeks to 50° at 14 weeks [41, 59].

According to reports, the greatest variation in CAx measurements occurs between 11 0/7 and 11 6/7 weeks of gestation, with a gradual decrease thereafter [59]. By the 12th week of gestation, the CAx is usually entirely levorotated [26, 41].

In a normal pregnancy, the CAx remains relatively stable after the 12th week of gestation, firmly establishing its position within the thoracic cavity [44, 60]. In the second and third trimesters, the CAx is characterized by a leftward deviation of the CAx by approximately 45° from the midline, with a range of variation of plus or minus 10-20° [58]. Beyond this range, deviations are viewed as disorders of the CAx and are typically the result of intracardiac or extracardiac abnormalities, such as congenital diaphragmatic hernia [26, 41, 48, 50, 60, 61].

**Fig. (2):**
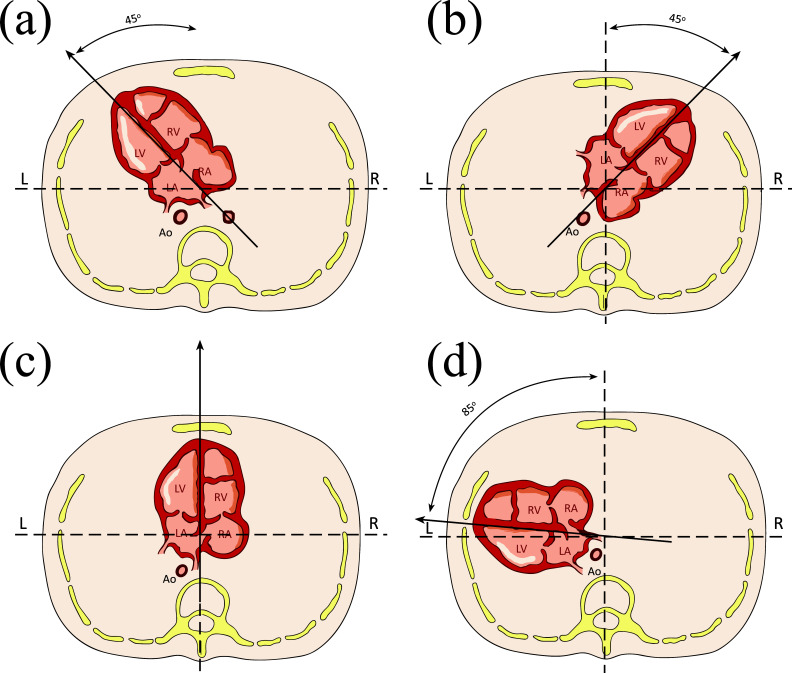
Four-chamber view of the fetal heart. Schematic drawing of a cross-section of the thorax at the level of the four-chamber view with an arrow tracing the long axis of the heart. Normal cardiac axis (characterized by a leftward deviation of the cardiac axis by approximately 45° from the midline, with a range of variation of plus or minus 10-20°) (**a**). Three distinct types of cardiac axis abnormalities: dextrocardia (the major axis of the heart is oriented to the right) (**b**); mesocardia (the longitudinal axis of the heart situated in the mid-sagittal plane) (**c**); and severe levocardia (leftward deviation of the cardiac axis beyond the angle previously mentioned) (**d**). LA, left atrium; RA, right atrium; LV, left ventricle; RV, right ventricle; Ao, aorta; L, left; R, right.

The available literature categorizes CAx abnormalities into three distinct types: dextrocardia, mesocardia, and severe levocardia (extreme left) (Fig. **2**). In fetal dextrocardia, the major axis of the heart is oriented to the right, as opposed to the typical leftward orientation (Fig. **2b**) [37, 62]. Mesocardia, on the other hand, is characterized by the displacement of the heart toward the midline of the thorax, with the longitudinal axis of the heart situated in the mid-sagittal plane (Fig. **2c**). Severe levocardia refers to a leftward deviation of the Cax beyond the angle previously mentioned (Fig. **2d**). The spectrum of cardiac diseases associated with these different types of CAx abnormalities is vast, and the clinical implications are diverse [37, 43, 48, 61, 62].

## 4 FETAL CARDIAC AXIS EVALUATION IN THE FIRST TRIMESTER

An assessment of the fetal chest's transverse plane at the level of the cardiac four-chamber view and the outflow tract views is usually performed during an obstetric ultrasound in the second trimester of pregnancy [43, 63]. The position and axis of the fetal heart can be evaluated accurately during fetal cardiac examination, even if the four-chamber view is not visualized satisfactorily [43, 64, 65].

Over the past ten years, with substantial technological advances in signal processing, the field of prenatal ultrasonography has significantly expanded. These advancements have substantially improved obstetrical practice and perinatal care [66, 67]. It is now feasible to perform high-quality fetal echocardiography, even during the first-trimester screening, allowing detailed investigation of fetal anatomy [19, 68-70].

In fact, the aim of making a diagnosis with the highest level of accuracy in early gestation is leading to growing research regarding not only the topic of first-trimester ultrasound, but also the determination of fetal CAx [41, 66, 71]. However, in contrast to the second trimester, little research has examined the normal values or the feasibility of the tool during the first trimester [41, 50, 57].

The success rate of CAx measurement increases with gestational age, and fetal heart screening during the first-trimester ultrasound can be challenging [41]. The difficulty in accurately measuring the CAx in the earliest gestations can be related to the small fetal size, increased heart frequency, active fetal movements, maternal obesity, an unfavorable position, and a lower amniotic fluid volume [41, 50, 66]. Furthermore, the fetal bones are poorly ossified, making adequate visualization difficult [41, 68]. The quality of the ultrasound equipment, as well as the level of expertise, training, and experience of the operators, are two other essential components [66].

**Fig. (3):**
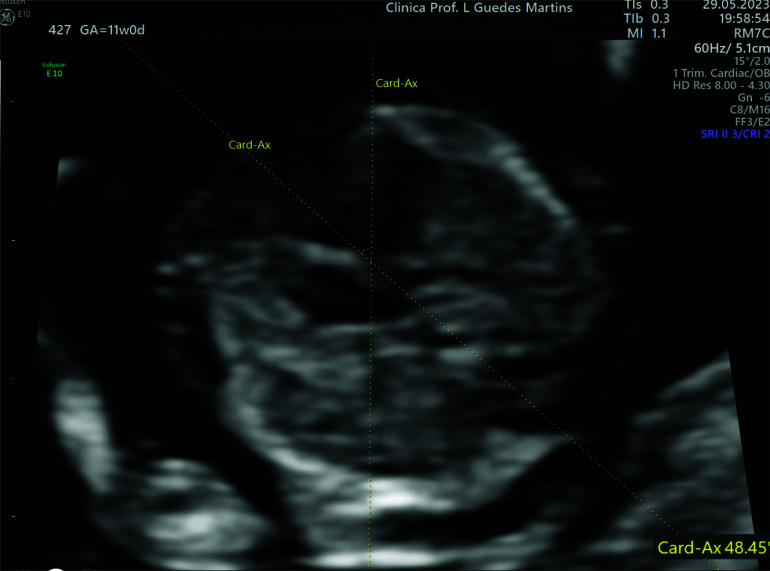
Cardiac axis measurement obtained in a fetus at 11 + 6 weeks of gestation. Cross-section of the thorax at the level of the four-chamber view demonstrating the normal cardiac axis. When the image of the chest can be obtained at this level, the cardiac axis can be measured as the angle between the line that bisects the thorax in the anteroposterior direction and the line tracing the heart's long axis. It can be noticed, in this image, the leftward deviation of the cardiac axis by approximately 48.45°.

However, according to data from published studies, sonographic assessment of the four-chamber view and the outflow tracts can be obtained in the first-trimester ultrasound with a high percentage of success (almost 100%) from as early as 11 weeks of gestation [21, 69, 72-75]. From the four-chamber view, it is often possible to ascertain the position of the CAx with a high degree of precision (Fig. **3**) [41, 59]. According to Jung *et al.* [31], a sonographer with one year of experience has a nearly 95% chance of detecting CAx during the first trimester. This allows the measurement of this tool even when it is difficult to see the detailed cardiac structures [41, 50, 67, 72, 76].

An axial sonographic image with a precise visualization of the cardiac chamber and one complete rib on each side of the fetal lateral chest wall can be used to create a four-chamber view [43]. When the image of the chest can be obtained at this level, the CAx can be measured as the angle between the line that bisects the thorax in the anteroposterior direction and the line tracing the heart's long axis (Fig. **3**) [31].

Since the fetal CAx only needs to be measured in the 4-chamber view during the routine surveillance procedure, specialized ultrasound knowledge is not necessary, and it is possible to assess the CAx without undergoing intensive training [59]. Thus, the use of the CAx as a screening tool in early gestation will not significantly extend the examination time or require additional sections [31, 57].

The predominant approach used in the initial studies involved transvaginal ultrasound [74, 77, 78]. However, recent studies, in particular those carried out after the 13^th^ week of pregnancy, have applied transabdominal ultrasound [79-81]. In general, prior to 12 weeks of pregnancy, transvaginal ultrasound is the preferred approach for fetal cardiac testing. Beyond 12 weeks, transabdominal ultrasonography is a reliable technique for fetal heart assessment [56].

Although CAx has been well defined in the second and third trimesters and is relatively constant at 45 ± 10-20°, some studies are now being conducted to define it at earlier gestational ages (Table **1**) [26, 31, 50, 59, 82].

However, there is a considerable degree of variability in the data that have been published related to the normal values of the CAx during early gestation. McBrien *et al.* [41] defined the normal value of CAx based on the gestational age; they reported that in early gestation, the CAx is oriented more to the midline of the thorax and then rotates to the left, changing from 25.1° at 8–9+6 weeks to 47.8° at 14–14+6 weeks (Table **1**). Sinkovskaya *et al.* [50] reported a normal variation in the CAx from 34.5° at 11 weeks to 56.8° at 13 + 6 weeks of gestation (Table **1**). On the other hand, Jung *et al.* [31] defined the mean CAx as 47.15° (with a standard deviation of 12.32) in their study (Table **1**). In contrast, Kesrouani *et al.* [59] revealed a different pattern for the CAx, reporting a decrease between 11 and 13+6 weeks of gestation. The mean value of CAx in this study was 48°, and it ranged from 39 to 60° (Table **1**) [59].

The small number of cases in previous studies, the technological challenges of cardiac imaging before 12 weeks of gestation, and variations in how patients were grouped by gestational age may all contribute to the discrepancies [59]. In addition, some of these studies identified limitations, such as the fact that they may not accurately reflect the general population, the use of exclusion criteria (*e.g*., maternal obesity), and the limited number of participants in early gestational age categories (Table **1**) [31, 41, 50].

To address these variations, additional research in larger studies is needed.

## 5 ABNORMAL FETAL CARDIAC AXIS AT EARLY GESTATIONAL AGE AND THE RISK FOR CONGENITAL HEART DEFECTS

Despite the use of high-frequency ultrasound probes that allow an early detailed assessment of high-quality cardiac images, the diagnosis of CHD in the first trimester still requires highly specialized training, and several cardiac defects may be missed [67, 80, 83-89]. It is still a developing practice performed by skilled professionals with a high level of expertise in early anomaly scanning and echocardiography [90, 91]. In fact, studies have reported detection rates for CHD of 10% or less when early echocardiography is carried out by less experienced operators [90, 92].

There are several challenging aspects related to the early diagnosis of CHD, particularly with respect to subtle and minor defects that are more difficult to observe [26, 93-95]. For example, certain forms of CHD, including hypoplastic left heart syndrome, are relatively easy to diagnose due to ventricular disproportion in the four-chamber view of an ultrasound examination [93-95].

However, prenatal diagnosis is significantly less common for other anomalies, such as transposition of the great arteries and ventricular septal defects, particularly small defects [93-95]. Another factor to consider is that some defects just evolve later in pregnancy or progress into more severe forms as the fetal heart grows (such as cardiac tumors, valvular stenosis as well as regurgitation, coarctation of the aorta, ductal constriction or aneurysm, cardiac tumors, complete heart block, and cardiac hypertrophy) [19, 76, 95-98]. Therefore, they may go unnoticed during an early fetal echocardiogram, and on these occasions, it is imperative to conduct follow-up examinations later in the pregnancy to accurately diagnose and monitor any potential cardiac anomalies [19, 76, 95-98].

Several indirect ultrasonographic markers, including increased NT [30, 99-101], tricuspid regurgitation [27, 98], and the detection of a reversed A-wave in the ductus venosus Doppler velocimetry [29, 102-104], have all been presented as important tools during the first trimester. These measurements are crucial in identifying high-risk pregnancies for CHD that may benefit from early fetal echocardiography, particularly in cases where the detection rate for cardiac anomalies is low throughout pregnancy [90, 91, 105].

Traditionally, ultrasound markers, such as increased NT have been applied not only for fetal chromosomal anomalies but also for the early detection of structural heart abnormalities [30, 99-101]. Nevertheless, it has been reported that only approximately 30% of CHD cases are detected using an NT of the 99^th^ percentile as a cutoff for fetal echocardiography referral [106]. Similarly, abnormal ductal flow has been associated with chromosomal abnormalities, cardiac defects, and adverse pregnancy outcomes [29, 102-104]. However, ductal flow examination requires operators with significant expertise due to common interference from adjacent vessels [105].

Despite advancements in prenatal screening, the first-trimester screening performance for CHD continues to be challenging, with average sensitivity values ranging from 54-74%, specificity values ranging from 94-100%, positive predictive values ranging from 93-100%, and negative predictive values ranging from 88-98% [26, 27, 29, 41, 50, 107-111].

CAx has been described in association with either intrinsic congenital heart diseases or extracardiac malformations (such as congenital diaphragmatic hernia) when assessed at the level of the four-chamber view [26, 48, 61, 65, 112]. Studies have reported a specificity of 98% and a sensitivity of 79.3% for CAx in detecting CHD when performed between 18 and 22 weeks of gestation [48, 65]. Additionally, CAx measurement in the first trimester has been suggested to have potential clinical applicability as a screening tool for CHD [26, 57].

According to research, a wide spectrum of different types of CHD have been described in association with an abnormal CAx during the first trimester [26, 57]. Specifically, researchers have found that leftward displacement of the CAx is the most common variation and is strongly associated with conotruncal anomalies (such as tetralogy of Fallot) and common arterial trunk [26, 50]. These kinds of anomalies are frequently missed when the four-chamber view is used alone [61, 65].

In the literature, it has been documented that fetuses with abnormal CAx can be observed in those with both small and large cardiac axes [26, 48, 57, 61]. Moreover, it has been reported that the nature of CHD can significantly influence the determination of the fetal right/left axis, with conotruncal abnormalities or complex cardiac malformations (such as univentricular hearts) demonstrating a higher prevalence [26, 48, 57, 61].

As an example, Sinkovskaya *et al.* [26] described an association between an abnormal CAx and coarctation of the aorta, Ebstein’s anomaly, transposition of the great vessels, and heterotaxy. The authors reported that between 11 0/7 and 14 6/7 weeks of gestation, an irregular deviation of the CAx is present in almost three-quarters of those with CHD [26]. Jung *et al.* [31] also demonstrated that an abnormal CAx is associated with a six-fold higher risk of CHD in the first trimester of pregnancy. The association between CHD and the presence of a left-axis deviation has also been examined in some studies [61, 65]. This shows the potential clinical utility of CAx in early gestation [26].

The detection of major cardiac defects *via* the measurement of the CAx at the level of the four-chamber view has revealed a sensitivity ranging from 50% to 74% [26, 31, 57, 113]. It has also been reported that CAx appears to have a better predictive value and may be a better method for detecting CHD than tricuspid valve regurgitation, the detection of abnormal ductal flow, and enlarged NT [26, 57].

Tetralogy of Fallot, coarctation of the aorta, hypoplastic left heart syndrome, and Ebstein anomaly of the tricuspid valve have been demonstrated to be the most frequent CHD related to a left-axis deviation, while a significant right deviation is most often related to a double-outlet right ventricle, an atrioventricular septal defect, and common atrium [50, 61, 113, 114]. Comparable results have been obtained during the second trimester and the early stages of gestation [48, 50, 115]. However, it has also been observed that some CHD may not exhibit an abnormal CAx [31, 50]. For example, it was reported that an isolated ventricular septal defect appears to have a minimal impact on the CAx due to its modest muscle involvement [31, 50].

These different types of CHD have been associated with distinct outcomes. As an example, morbidity and mortality rates in patients with hypoplastic left heart syndrome remain relatively high, and despite its relatively low incidence, it accounts for about 23% of cardiac deaths in the first week of life [116]. In addition, Tetralogy of Fallot is a very complex and cyanotic form of CHD, and its prognosis remains significantly poor, particularly for those who have not undergone surgical repair [117]. Both Tetralogy of Fallot and hypoplastic left heart syndrome have been associated with significant leftward deviation of the CAx [41, 50,26]. Specifically, cases of Tetralogy of Fallot were reported to have the most abnormal CAx measurements (over the 90^th^ percentile) [41]. In contrast, the ventricular septal defect is a common CHD with a distinct prognosis [118]. Often asymptomatic, it tends to close spontaneously during infancy [119]. Normally, patients with minor ventricular septal defects have a favorable prognosis and a low incidence of cardiac complications even without intervention [118, 119]. Therefore, the potential consequences of failing to detect this anomaly appear to be minor.

Jung *et al.* [31] reported that CAx should be taken into consideration when combined with other sonographic findings and not alone for the screening of CHD [31]. They reported that none of the fetuses diagnosed with CHD or aneuploidy in their study exhibited a CAx in the absence of additional abnormal sonographic observations [31]. Furthermore, the authors reported that the measurement of CAx in combination with other variables, including the measurement of NT, blood flow in the ductus venosus, and the presence of tricuspid regurgitation, had a higher specificity than the measurement of the CAx alone in the first trimester (93.2%, 100%, 99.3%, and 72.3%, respectively) [31]. Therefore, the performance of the CAx in combination with other first-trimester ultrasound findings may allow for the optimization of this marker and improve the detection of CHD [31].

The presence of cardiac abnormalities on prenatal sonography also raises suspicion of a wide range of genetic syndromes and fetal aneuploidy [95]. According to studies, the prevalence of aneuploidy can be as high as 22-32% in fetuses with CHD [120, 121]. Therefore, the identification of an abnormal CAx in the first trimester may not only improve the detection of CHD but also increase the likelihood of identifying fetal aneuploidy [31, 57].

Contrary to NT, investigations have also revealed that CAx is effective in detecting CHD in fetuses with both normal and abnormal karyotypes, which is a significant advantage when compared to NT [26].

Thus, the detection of CAx may have the potential to enhance the early detection of CHD that would otherwise have only minor variations or not even be detectable in the four-chamber view [26, 61, 65]. It may be helpful in defining a population at risk for fetal CHD that warrants further sonographic evaluation with a detailed anatomical examination. This may allow for the optimization of perinatal management, with appropriate counseling of patients and enhanced intrauterine treatment [50, 60, 61, 82, 113, 122, 123].

However, the available literature on this topic is still limited, and there are relatively few studies published in the literature with some heterogeneous results.

## 6 ABNORMAL FETAL CARDIAC AXIS AT EARLY GESTATIONAL AGE AND THE RISK OF EXTRACARDIAC ABNORMALITIES

The presence of a pathologic intracardiac or extracardiac condition can change the fetal CAx and/or its position in the chest [65, 124]. Various disorders, such as cardiac malformations, diaphragmatic eventration, abdominal wall deformities, pericardial and pulmonary tumors, fetal lung hypoplasia or agenesis, pleural diseases, and diaphragmatic hernia, have been reported to contribute to heart displacement and abnormalities in the fetal CAx [55, 60, 65, 125-129]. In general, an improper position of the heart indicates an extracardiac problem that may include abnormalities of the thoracoabdominal wall and intrathoracic pathologic findings, and an abnormal CAx is indicative of a wide spectrum of intracardiac structural anomalies [26, 60, 61, 65, 126, 128, 130-132].

Nevertheless, there is some evidence that anomalies in the CAx, with reported cases of either dextrocardia or levocardia, may also be indicative of underlying extracardiac defects [48, 62, 65, 115, 124, 129, 131, 133-137]. Specifically, congenital cystic adenomatoid malformation, pulmonary agenesis, and diaphragmatic abnormalities (including diaphragmatic eventration or congenital diaphragmatic hernia) have been identified as conditions associated with an abnormal CAx [136-138]. In cases of space-occupying lesions, the fetal cardiomediastinal shift is generally caused by the intrathoracic lesion compressing the fetal heart, resulting in an abnormal CAx [51-54].

Although there are relatively few studies published on this association at earlier gestational ages, Tuzovic *et al.* [139] concluded that abnormalities of the CAx and/or cardiac position are associated with fetal congenital lung lesions and present in 45% of fetuses before 24 weeks of gestation and in 38% of fetuses between 24 and 32 weeks [136, 137, 139]. Furthermore, abnormalities in the CAx have also been reported in fetuses with a congenital diaphragmatic hernia during the first trimester, including cases of dextrocardia [136, 137].

In terms of anomalies in the abdominal wall, an abnormal CAx was also described in cases of omphalocele and gastroschisis (the two most common types of abdominal wall malformations that result in the herniation of abdominal structures), including at earlier gestational ages [48, 55, 65, 133, 135-140].

In fact, a few prospective and retrospective studies have published a correlation between fetuses exhibiting a left-axis deviation and cases of omphalocele, which refers to ventral abdominal wall defects where the protruded organs are covered by a protective membrane [55, 65, 115, 133, 140]. An abnormal CAx was described in approximately 50% of fetuses diagnosed with this condition [55, 139]. Furthermore, during the first trimester, a case of sirenomelia was reported, demonstrating an association between dextrocardia and omphalocele [134].

This association can potentially be elucidated by considering the simultaneous events that take place during embryological development, particularly the events related to the embryological disorders affecting the developing heart and the anterior abdominal wall. At approximately 8 weeks of gestation, the fetal heart undergoes both vertical and horizontal rotations; simultaneously, the bowel enters the umbilical cord, undergoes rotation on its own axis, and subsequently returns to the abdomen [55]. According to this theory, since these systems develop in parallel, an adverse event occurring during this stage of embryogenesis may eventually lead to the development of an omphalocele and an abnormal CAx, even in the absence of CHD [55, 65, 133]. As an example, a study conducted by Smith *et al.* [65] reported six cases of fetuses with left-axis deviation and omphalocele, of which only three had an abnormal CAx [65].

Moreover, gastroschisis represents an abdominal wall defect characterized by the absence of a protective membrane, resulting in the exposed protrusion of abdominal organs, and has also been associated with an abnormal CAx [55, 141]. Several etiological factors have been suggested in the literature, such as disturbances in mesenchymal somatic migration and ischemia of the abdominal wall caused by the involution of the right umbilical vein or vitelline artery [55]. However, these proposed mechanisms do not appear to have an impact on embryo cardiac development and are unable to explain the findings of abnormal CAx in the reported cases [55]. Additional research in larger studies that include fetuses with abdominal wall abnormalities is needed, particularly at earlier gestational ages.

The detection of an abnormal CAx may have the potential to reflect underlying cardiac and extracardiac defects, which is crucial to allow appropriate prenatal counseling as well as the best management course plan for those affected cases, considering that these anomalies can substantially impact prognostic outcomes [126].

## 7 DISCUSSION

CHD represents the most prevalent type of congenital malformation resulting from abnormal fetal heart development and constitutes a significant contributor to morbidity and mortality; therefore, a comprehensive multidisciplinary management approach is needed [1, 12, 132, 142].

Numerous studies have demonstrated that increased investment in detecting fetal anomalies in early gestation will not only predict postnatal prognosis but can also provide other benefits, such as time for genetic evaluation, opportunities for informed prenatal counseling for parents, potential treatment options without delay, relocation of the family to centers properly equipped or even the possibility of pregnancy termination [50, 60, 61, 82, 113, 122, 123]. The ability to diagnose CHD earlier and with greater precision may have the potential to improve long-term outcomes, reduce healthcare costs, and provide additional time for necessary arrangements in both the economic and family spheres [68, 101]. Consequently, numerous efforts are currently being made to diagnose structural cardiac defects in fetuses during the first trimester of pregnancy [19, 90, 91, 101, 105].

As a result of significant technological advancements, the diagnosis of CHD has increased substantially over the past two decades and is currently in a continuous growth phase [19, 143]. Nevertheless, the accurate identification of fetal anomalies, particularly structural heart defects, during the first trimester remains a challenging task within routine screening programs, requiring extensive and specialized training [67, 80, 83-89].

There are several difficulties that ultrasound technicians usually face regarding the early diagnosis of CHD, including the small dimensions of the fetal heart, increased heart frequency, and minor defects that are difficult to see and can potentially be missed [26, 68, 93-95]. Consequently, there is growing interest in research related to systematic assessment of first-trimester ultrasound markers for CHD, including measurement of the fetal CAx, which is becoming more widespread [26, 57].

Sonographic assessment of the four-chamber view during the routine surveillance procedure allows the measurement of the CAx with a notably high success rate from as early as 11 weeks of gestation [21, 31, 41, 69, 72-75, 132]. However, although CAx has been well defined in the second and third trimesters, less research has been published at earlier gestational ages. In fact, there is considerable variability in the reported results concerning the normal values of the CAx [31, 41, 50]. The technological challenges of cardiac imaging and the low numbers of studied fetuses in early gestational age categories have been identified as potential limitations contributing to this discrepancy [31, 41, 50, 59]. Thus, future investigations necessitate larger-scale population-based studies to provide more comprehensive insights and knowledge of normal changes in the CAx and its reference values in early pregnancy.

Research studies have provided evidence that abnormal CAx can serve as a reliable and powerful tool for detecting CHD during early gestation [26, 50]. It was reported that the establishment of the fetal right/left axis is significantly influenced by the type of CHD, with a higher prevalence in conotruncal abnormalities or complex cardiac malformations [26, 48, 57, 61]. Three distinct types of CAx abnormalities (dextrocardia, mesocardia, and severe levocardia) have been described, and they have all been associated with a wide range of CHD [26, 48, 57, 61].

Moreover, some published studies have established an association between abnormal CAx and extracardiac abnormalities, including at earlier gestational ages, such as congenital cystic adenomatoid malformation, fetal congenital lung lesions, congenital diaphragmatic hernia, and omphalocele [26, 48, 61, 65, 112].

The assessment of the four-chamber view of the heart alone may not be sufficient to diagnose certain anomalies, as highlighted in previous studies [61, 65]. Therefore, some studies assert that the CAx is of higher value as a marker for certain abnormalities that would otherwise not be fully detectable with the four-chamber view [61, 65]. Thus, an abnormal CAx may have potential clinical applicability for identifying pregnancies at high risk for CHD that could potentially benefit from early fetal echocardiography with an extended and detailed anatomical evaluation [90, 91, 105].

However, it was also observed that some CHD may not exhibit an abnormal CAx (such as an isolated ventricular septal defect due to its modest muscle involvement) [31, 50]. It was also reported that the CAx should be considered along with other sonographic findings to improve the specificity [31].

Nevertheless, despite some existing reports on the topic, further investigations are crucial to fully understand the potential value of CAx measurement in detecting CHD in early gestation. Large-scale population studies addressing this association are limited, and clear management protocols are yet to be established.

The inclusion of more data reporting and validating the clinical applicability of CAx measurement will emphasize the importance of including this tool as a standard component of first-trimester fetal ultrasound examinations.

## CONCLUSION

In conclusion, fetal CAx represents a promising and strong tool and may provide the opportunity to detect a wide range of CHD in early pregnancy and improve the management and perinatal prognosis of affected fetuses [50, 60, 61, 82, 112, 123, 123]. Studies conducted during the first trimester have demonstrated promising outcomes concerning the potential applicability of CAx measurement in fetuses with CHD, as well as those with extracardiac and chromosomal anomalies [31, 57, 65].

Nevertheless, further research efforts in the field of first-trimester assessment need to be intensified to optimize the clinical utility of CAx measurement with clear management protocols.

## Figures and Tables

**Table 1:** Studies on the evaluation and values of the fetal cardiac axis in early gestation (from 2010, newer to older). CAx, cardiac axis.

Study	Total n	Type of Article	GA, Weeks	Population	Ultrasound Approach	Cardiac Axis	Limitations	Conclusion
Kesrouani *et al.*, 2021 [59]	100	Prospective	11.0-13.6	One hundred pregnant patients present for their first-trimester screening ultrasound	Level of the four-chamber view. Transabdominal approach was used in 93% of the cases. A combined transabdominal and transvaginal approach in 7%.	48±5,2°, (ranging from 39° to 60°)	Did not have a group with an abnormal fetal CAx.	CAx was measurable in all the cases. The mean CAx was 48±5.2°. CAx tends to decrease between 11 and 13+6 weeks of gestation.
Jung *et al.*, 2020 [31]	142	Retrospective	11.0–13.6	Pregnant women who visited the Prenatal Diagnosis Clinic of the Chung-Ang University Hospital (high-risk pregnancy center).	Level of the four-chamber view	14.80° to 75.70°	May not represent the general population (hospital with a high proportion of high-risk pregnancy cases).	In the group of normal fetuses, the cardiac axis ranged from 14.80° to 75.70°.
Adekola *et al.*, 2016 [82]	324	Prospective	11.0-15.0	Women with singleton pregnancies. Exclusion criteria included pregnancies complicated by severe maternal illness and fetal demise.	Level of the four-chamber view	48.1 ± 7.1° (11–15 weeks)	The study population consisted of only one ethnicity. To validate the absence of cardiac anomalies, delivery and neonatal assessment records for 23% of the fetuses were not available for review.	In normal fetuses, the CAx remains stable between 11 and 15 weeks of gestation and it is less levorotated at 18–22 weeks. Maternal obesity had no effect on the fetal cardiac axis.
Sinkovskaya *et al.*, 2015 [26]	197	Retrospective multicenter case–control study	11.0-14.6	Medical records of all pregnant women who presented for first-trimester screening between January 2005 and June 30, 2011. Cases involving pregnancy terminations, miscarriages, or loss of follow-up prior to 18 weeks of gestation were excluded from the study.	Level of the four-chamber view	24° to 68° (mean 44.5 ± 67.4°)	A case-control study design did not allow for estimating the diagnostic value of the cardiac axis measurement for the general population.	In the control group (cases with normal ultrasound findings and known uncomplicated pregnancy outcome), the CAx ranged from 24° to 68°.
McBrien *et al.*, 2013 [26]	166	Prospective	8.0-14.6	Healthy women with singleton pregnancies	Level of the four-chamber view. Transvaginal approach was predominantly used at < 10 weeks’ gestation and a transabdomin al approach at >10 weeks.	25.5 ± 11.5° (8 + 0 to 9 + 6 weeks) 40.4 ± 9.2° (10 + 0 to 11 + 6 weeks) 49.2 ± 7.4° (12 weeks) 50.6 ± 5.7° (13 weeks) 48.6 ± 7.3° (14 weeks)	The technological challenges of cardiac imaging. Small numbers in the earliest gestation groups.	CAx is midline at 8 weeks and levorotates in the late first trimester. By 12 weeks’ gestation, CAx is established and remains stable until at 14 + 6 weeks.
Sinkovskaya *et al.*, 2010 [50]	94	Prospective observational cohort study	11.0-14.6	Women, ≥ 18 years and with singleton pregnancies. Exclusion criteria included maternal obesity [body mass index (BMI) ≥ 30].	Level of the four-chamber view	34.5 to 56.8◦ (mean 47.6 ± 5.6◦)	Exclusion criteria included maternal obesity [body mass index (BMI) ≥ 30].	CAx measurement is possible in the first and early second trimesters.

## LIST OF ABBREVIATIONS

bHLH: Basic Helix-Loop-Helix
CHD: Congenital Heart Defects
CAx: Cardiac Axis
GATA5: GATA Binding Protein 5
GATA6: GATA Binding Protein 6
NT: Nuchal Translucency
PITX2: Paired-like Homeodomain Transcription Factor 2
